# 2751. Cefiderocol Use in Treating Patients with Confirmed *Stenotrophomonas maltophilia* Infections in US Hospitals During January 2020 - June 2022

**DOI:** 10.1093/ofid/ofad500.2362

**Published:** 2023-11-27

**Authors:** Bin Cai, Sean T Nguyen, Jennifer D Copeland, Hyun Jin Song, Christine M Slover

**Affiliations:** Shionogi Inc, Florham Park, New Jersey; Shionogi Inc., Florham Park, New Jersey; Shionogi, Inc., Houston, Texas; Genesis Research, Gainesville, Florida; Shionogi Inc., Florham Park, New Jersey

## Abstract

**Background:**

*Stenotrophomonas maltophilia* (SM) infections have been associated with high mortality rates (21% to 69%), particularly in critically ill patients because of SM inherent resistance to many antibiotics, including carbapenems. This study describes cefiderocol (CFDC) treating hospitalized patients infected with SM in the US.

**Methods:**

This retrospective observational study used January 2020-June 22 Premier Healthcare Database. Inclusion criteria are age ≥18 years, non-COVID, a positive SM culture without other Gram-negative pathogens identified within ±3 days and received CFDC ≥3 days on or after first SM culture. This study describes the patient characteristics, cefiderocol use pattern, and overall, 14-day and 28-day in-hospital all-cause mortalities (IHACM) with 95% confidence intervals (CI). First SM culture is the Index culture.

**Results:**

Of 19 patients meeting the inclusion criteria, median age was 62 years with an interquartile range (IQR) of 56-76 years, 57.9% were male, 63.2% came from home, and 89.5% were admitted via emergency room or urgent care. Top comorbidities at admission were renal disease (68.4%), congestive heart failure (52.6%), chronic pulmonary disease (31.6%), and peripheral vascular disease (31.6%). Over 68% of patients were in the ICU, with the same rate of receiving both mechanical ventilation and vasopressor support at the time of index culture. Also 68.4% of index cultures were from respiratory and 10.5% from blood. CFDC was initiated ≤5 days of index culture in 8 (42.1%) patients, 6-20 days in 9 (47.4%) patients, and >20 days in 2 (10.5%) patients. Median days on CFDC was 8 days (IQR: 4-12), longer for patients who started CFDC later. All 19 patients received other antibiotics prior to CFDC. Twelve (63.2%) patients received other antibiotics during CFDC treatment, and the most common antibiotics were levofloxacin (50.0%), eravacycline (50.0%), cefepime (33.3%), or minocycline (33.3%). Median of hospital stays after index culture was 18 days (IRQ:8-30). Crude overall IHACM was 36.8% (95%CI: 15.8%-58.5%). Crude 28-day IHACM from index culture was 21.1% (95%CI: 2.7%-39.4%).

**Figure:**
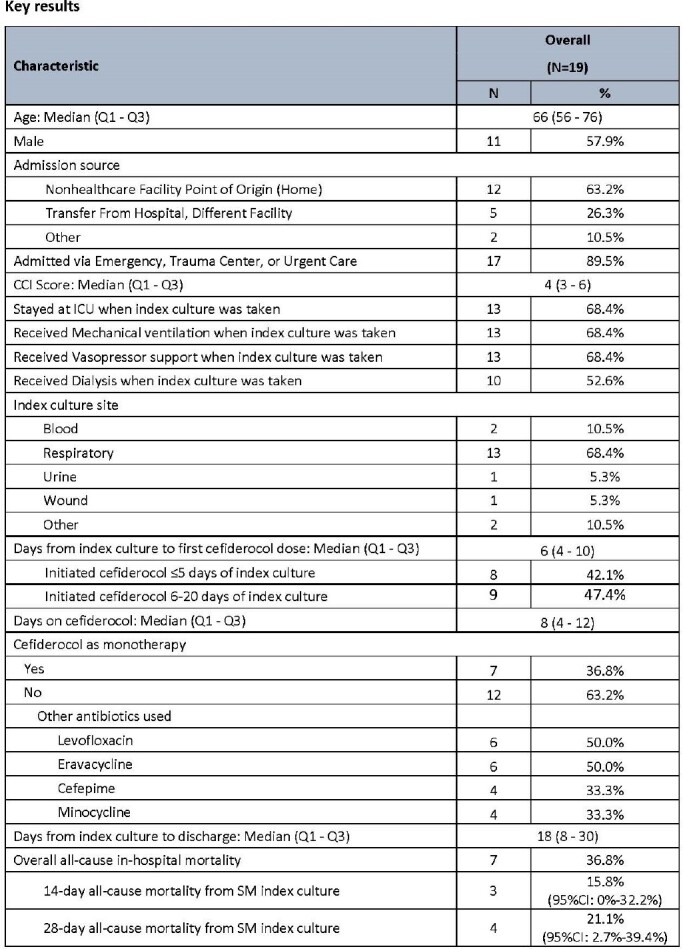

**Conclusion:**

SM is a rare Gram-negative pathogen with limited treatment options. CFDC offers an additional treatment option against this pathogen. Further investigation is warranted.

**Disclosures:**

**Bin Cai, MD, PhD**, Shionogi Inc.: Shionogi employee **Sean T. Nguyen, PharmD**, Shionogi: Employee|Shionogi, Inc: Employee **Jennifer D. copeland, MS**, Shionogi, Inc.: Employee (Medical/Micro) **Christine M. Slover, PharmD**, Shionogi,INC: Employee

